# Arthroscopic Discopexy Techniques for Articular Disc Displacement: A Systematic Review and Meta-Analysis

**DOI:** 10.3390/jcm14228046

**Published:** 2025-11-13

**Authors:** Shinnosuke Nogami, Phasathorn Jewrasumnuay, Kensuke Yamauchi

**Affiliations:** Division of Oral and Maxillofacial Reconstructive Surgery, Tohoku University Graduate School of Dentistry, 4-1 Seiryo-machi, Aoba-ku, Sendai 980-8575, Miyagi, Japan; phasathorn.jewrasumnuay.s7@dc.tohoku.ac.jp (P.J.);

**Keywords:** temporomandibular joint, arthroscopic discopexy, disc repositioning, anterior disc displacement, suture anchor fixation

## Abstract

**Background/Objectives**: Anterior disc displacement (ADD) is a common temporomandibular joint (TMJ) disorder and may progress to internal derangements. Although arthroscopic discopexy (minimally invasive disc repositioning with fixation) has been adopted, suggested techniques and pooled outcomes have not been comprehensively synthesized. The aim of the present study was to summarize the effectiveness of arthroscopic discopexy in treating patients with disc-related TMJ disorders. **Methods**: This systematic review and meta-analysis followed Cochrane guidance and PRISMA 2020 protocol. Four databases were searched through 1 September 2025. A total of 26 studies were included in this review. Nine studies met the eligibility criteria for meta-analysis and were pooled. The remaining 17 studies were narratively described, focusing on surgical characteristics. Continuous outcomes (MIO, pain score (VAS 0–10)) were pooled as mean differences (MD) with 95% confidence intervals (CIs), and joint sounds were synthesized as dichotomous outcomes. Analyses and heterogeneity were performed in RevMan 5.4. Certainty was graded with GRADE. (PROSPERO: CRD420251145229). **Results**: 1086 TMJs were analyzed. Arthroscopic discopexy significantly improved MIO, pain, and joint sounds at all time points. The MD of MIO was 10.58 mm (95% CI: 4.46 to 16.70; *p* ≤ 0.001), 9.83 mm (95% CI: 4.09 to 15.57; *p* ≤ 0.001), and 13.06 mm (95% CI: 4.40 to 21.72; *p* ≤ 0.001), respectively. The MD of the pain score was −4.36 (95% CI: −6.89 to −1.82; *p* ≤ 0.001), −3.91 (95% CI: −6.23 to −1.59; *p* ≤ 0.001), and −4.56 (95% CI: −7.81 to −1.31; *p* < 0.01), respectively. At 12 months, joint sounds were less frequent than preoperatively (OR = 0.07; 95% CI: 0.01 to 0.37; *p* < 0.01). Overall, the certainty of evidence according to the GRADE approach was rated as low. Therefore, the results should be interpreted with caution, as high heterogeneity was observed across the three follow-up time points and the included studies were observational. **Conclusions**: These findings underscore the significance of arthroscopic discopexy in enhancing TMJ function and alleviating symptoms. Current evidence, characterized by a low risk of bias and low certainty, supports the advantage of arthroscopic discopexy. Due to the observational evidence base and heterogeneity, high-quality randomized trials conducted under standardized treatment protocols and with longer follow-up are needed.

## 1. Introduction

The temporomandibular joint (TMJ) plays a fundamental role in maintaining essential functions for human survival, including occlusion and the masticatory system. Similar to the knee joint, which bears body weight, the TMJ is responsible for absorbing and distributing loads generated during occlusal contact and mastication. Mastication imposes mechanical stress on the TMJ, which the joint must withstand. It exhibits highly specialized and complex anatomical characteristics. However, excessive and repetitive loading, as seen in parafunctional habits such as bruxism, can exceed the adaptive capacity of the joint, leading to TMJ arthritis, internal derangements (IDs), and ultimately temporomandibular joint disorders (TMDs). TMDs represent one of the most common TMJ pathologies, with an estimated global prevalence of approximately 34% [1]. The TMJ is most commonly attributed to anterior disc displacement (ADD), which has been reported to result in reduced maximal mouth opening and impairment of masticatory efficiency [2,3]. In patients with dentofacial deformities, TMD is reported to occur more frequently in individuals with Class II malocclusion or facial asymmetry [4]. Of particular concern is progressive condylar resorption (PCR), which has been documented following orthognathic surgery in patients with Class II malocclusion [5,6,7]. The etiology of PCR remains unclear. However, excessive mechanical stress and the influence of sex hormones have been suggested as contributing factors [8,9,10]. Our previous studies have demonstrated, through animal models, the association of these factors with PCR [11,12,13]. Importantly, we further identified that patients with PCR often present with ID before orthognathic surgery [14], with anterior disc displacement without reduction (ADDwoR) being particularly prevalent [15,16,17,18,19,20]. These findings highlight the clinical relevance of pre-existing ID in patients with mandibular retrognathism, suggesting that failure to address such pathology may predispose patients to postoperative PCR. Therefore, careful assessment and management of ID—especially ADDwoR—should be considered an integral component of preoperative evaluation and treatment planning in orthognathic surgery. Therapeutic approaches for ADDwoR include physical therapy, occlusal splint therapy, arthrocentesis, and disc repositioning procedures [19,21,22].

Clinical intervention is typically necessary when patients experience TMD, characterized by joint pain or limited mouth opening. Treatment often progresses stepwise. First, physical therapy begins, followed by splint therapy. Arthrocentesis is used if needed. The role of disc repositioning remains controversial due to a lack of high-quality and long-term evidence. Narrative reviews have mentioned disc repositioning, but few systematic reviews have evaluated its outcomes for ADDwoR. Moreover, for severe ADD, the strong scientific evidence is limited to open surgery. Nonetheless, numerous reports show favourable postoperative outcomes with arthroscopic discopexy. We therefore hypothesized that arthroscopic discopexy would improve MIO, reduce pain, and diminish joint sounds at 6, 12, and 24 months compared with preoperative values.

## 2. Materials and Methods

The study protocol of this systematic review and meta-analysis was conducted in accordance with the Cochrane Handbook and reported following the Preferred Reporting Items for Systematic Reviews and Meta-analysis (PRISMA) 2020 statement [23,24]. This review was registered in the International Prospective Register of Systematic reviews (PROSPERO) before data extraction began (registration number: CRD420251145229). Ethical approval was not required for this review, as it involved no direct access to identifiable patient information or direct approach with participants.

### 2.1. Aim and Objectives

The goal of this review was to summarize the effectiveness of arthroscopic discopexy for patients with anterior disc displacement (ADD), both with and without reduction. We assessed postoperative pain, MIO, and joint sounds improved at 6, 12, and 24 months and compared them with preoperative values. We ensured that all results were reported in standardized units/scales. The review protocol was prospectively registered in the PROSPERO database (ID: CRD420251145229).

### 2.2. Research Question

How does arthroscopic discopexy improve TMJ function and alleviate symptoms at 6, 12, and 24 months after treatment?

### 2.3. Eligibility Criteria and Search Strategy

Two researchers (N.S. and J.P.) looked for studies about this topic published before 1 September 2025 from four electronic databases (PubMed, Embase, Scopus, Web of Science). All search strategies were provided in Supplementary Table S1. They followed review guidelines to determine search terms and select the types of studies to include or exclude. Details about the search terms and the criteria for inclusion and exclusion are shown in Table 1 and Table 2.

### 2.4. Data Extraction and Management

After collecting the data, duplicate records were removed using EndNote 21 and Rayyan [25] (version 1.4.3). Screening was conducted in two stages: titles and abstracts were screened first, followed by a full-text review. Two researchers (N.S. and J.P.) worked independently. Disagreements were resolved by discussion, with final decisions by the third author (Y.K.). The full texts of potentially eligible articles were reviewed against inclusion and exclusion criteria (Table 2). All exclusion reasons were recorded. If a study described a relevant surgical procedure, it was also reviewed. Articles that met the criteria were included in the meta-analysis. Articles that described surgical techniques were excluded from meta-analysis but retained for qualitative discussion. Two researchers (N.S. and J.P.) extracted general characteristics of articles. Another researcher (Y.K.) verified the extracted data. Extracted data included: first author, publication year, study design, numbers of participants and TMJs, participant characteristics, suture material, disc repositioning and fixation technique, anchorage location or material, co-interventions, pain score (measured by visual analogue scale (VAS) 0–10; 0 representing no pain and 10 representing the worst pain; mean ± SD), maximal interincisal opening (MIO, mean ± SD), and improvement in joint sounds (number of patients with or without clicking, popping, or crepitation). Data that were unclear or missing were noted and excluded from evaluation.

### 2.5. Assessment of Research Quality

Two researchers (N.S. and J.P.) assessed the quality of the studies, using checklists from the National Heart, Lung, and Blood Institute (NHLBI) Quality Assessment Tool (Quality Assessment Tool for Before-After (Pre-Post) Studies with No Control Group and Quality Assessment Tool for Case Series Studies) [26]. Another researcher (Y.K.) randomly checked some of the ratings. If they disagreed, they discussed it until they reached an agreement. Y.K. made the final decision.

### 2.6. Data Synthesis and Statistical Analysis

The meta-analysis was conducted using three time points (6-month, 12-month, and 24-month follow-up) and three variables (pain score, MIO, and joint sounds), comparing baseline (pre-surgery) with post-surgery outcomes. Effect sizes were calculated as mean differences (MD) with standard deviations (SD) for pain and MIO scores, and as dichotomous outcomes for joint sounds. A 95% confidence interval (CI) was set at α = 0.05. Pairwise comparisons between pre- and post-surgery were evaluated using MD. Heterogeneity was assessed by the chi-square test (χ^2^) and the I^2^ statistic, with levels interpreted as low (<25%), moderate (25–50%), and high (≥50%). Forest plots for each outcome at each time-point model were generated to illustrate the pooled results. Statistical analyses were performed in StataNow19 (StataCorp LLC, College Station, TX, USA; 2025). Significance was defined as a *p*-value of less than 0.05, highly significant as a value of less than 0.01, and the highest significance as a value equal to or less than 0.001. The quality of the evidence was evaluated using the GRADE (Grading of Recommendations Assessment, Development, and Evaluation) framework and categorized as high, moderate, low, or very low [27].

## 3. Results

### 3.1. Study Selection

**Figure 1:**
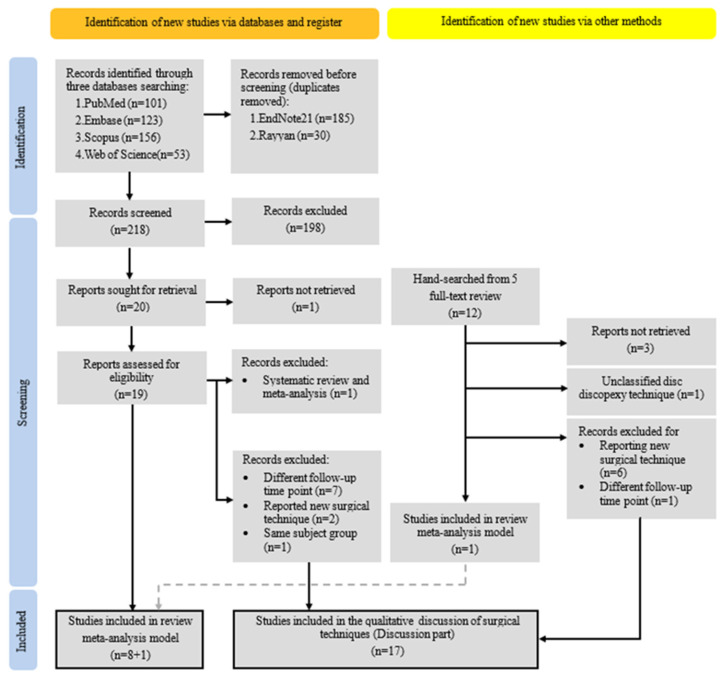
PRISMA 2020 process flow diagram illustrates the literature selection process.

Following the PRISMA 2020 guidelines, the screening process is summarized in the PRISMA flow diagram (Figure 1). From four electronic databases, 433 articles were initially identified. After 215 duplicated articles were removed, 218 records remained for title and abstract screening. Twenty articles that met the inclusion criteria (Table 2) were assessed in the full screening process. Among them, only eight studies with accessible full texts and one from hand-searched articles that clearly reported postoperative pain, MIO, or joint sounds at 6, 12, or 24 months after surgery were included in the meta-analysis models. Ten excluded articles and seven additional full-text articles identified through hand-searching, all of which described arthroscopic disc repositioning and anchor fixation techniques, were included in the qualitative discussion of surgical techniques.

### 3.2. Characteristics and Quality of the Included Studies

A total of 1086 temporomandibular joints (TMJs) with ADD, based on clinical characteristics and magnetic resonance imaging (MRI), were included and analyzed at postoperative three-time points. The functional parameters pain score (VAS, 0–10; mean ± SD), maximal interincisal opening (MIO; measured as the maximum distance between the incisal edges of the upper and lower incisors during painless mouth opening, in millimetres), and the presence of joint sounds were evaluated and recorded (present or absent). Descriptive information and clinical outcome of the studies included in each meta-analysis model are presented in Table 3 and Table 4. Overall, postoperative trends of surgical outcomes showed that arthroscopic discopexy techniques were associated with improved MIO, reduced pain, and concomitant reductions in joint sounds. An exception was the study by McCain et al. (2015) [16], which reported higher postoperative pain compared with preoperative levels.

**Figure 2:**
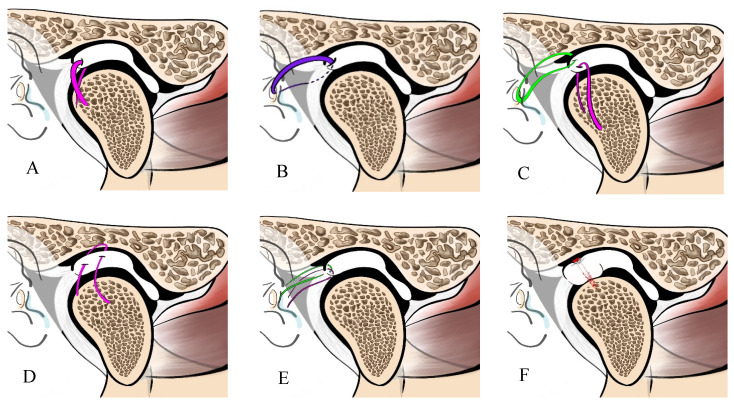
Summary of arthroscopic discopexy techniques, focusing on suture methods and anchorage sites. (**A**) Single suture discopexy technique (at the posterior lateral corner of the disc-condyle). (**B**) Single suture discopexy technique (at bilaminar zone or retrodiscal zone). (**C**) Double-suture arthroscopic discopexy. (**D**) Posterior double-pass suture technique (the needle penetrating the disc from lateral to medial in the posterior zone). (**E**) Double horizontal mattress-like suturing technique (Yang’s technique). (**F**) An arthroscopic disc repositioning using a resorbable pin.

Six different TMJ disc fixation techniques, derived from twenty-five research articles, are summarized in Table 5 and Figure 2.

### 3.3. Research Quality Assessment

Table 6 and Table 7 illustrate the quality of each study, assessed with the NHLBI tool and categorized by study design. Most were rated as good quality, except for the research by McCain et al. in 2015, which was rated as poor quality mainly because only the surgical outcomes of the successful group were reported; other reasons contributing to this rating are listed in Table 6 [16].

### 3.4. Synthesis of Results

A total of 1086 TMJs from 811 patients and nine studies were divided into three main meta-analysis models, depending on the outcome variable (MIO, pain score, joint sounds), and subgrouped into three postoperative observational time points (6 months, 12 months, and 24 months). Three variables (MIO, pain score, and joint sounds) were evaluated before and after surgery (Table 4).

**Figure 3:**
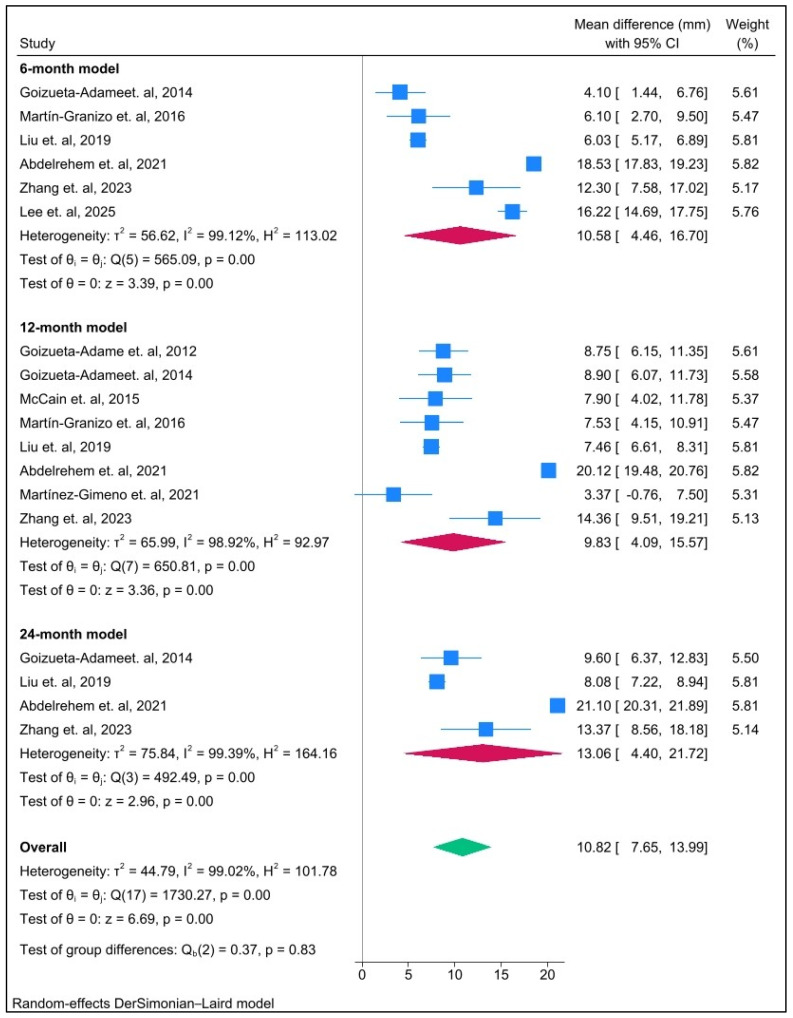
Forest plot illustrating postoperative maximal interincisal opening (MIO; mm) compared with pre-surgery (baseline) across different follow-up time points.

Arthroscopic disc repositioning and anchor fixation techniques demonstrated a significant improvement in postoperative MIO at all time points. The most significant effect was observed in the short-term (6-month) follow-up period (MD = 10.58 mm; 95% CI: 4.46 to 16.70; *p* ≤ 0.001; I^2^ = 99%), the intermediate-term (12-month) follow-up period (MD = 9.83 mm; 95% CI: 4.09 to 15.57; *p* ≤ 0.001; I^2^ = 99%), and in the long-term (24-month) follow-up period (MD = 13.06 mm; 95% CI: 4.40 to 21.72; *p* ≤ 0.001; I^2^ = 99%). Heterogeneity was high across the three time points (Figure 3).

**Figure 4:**
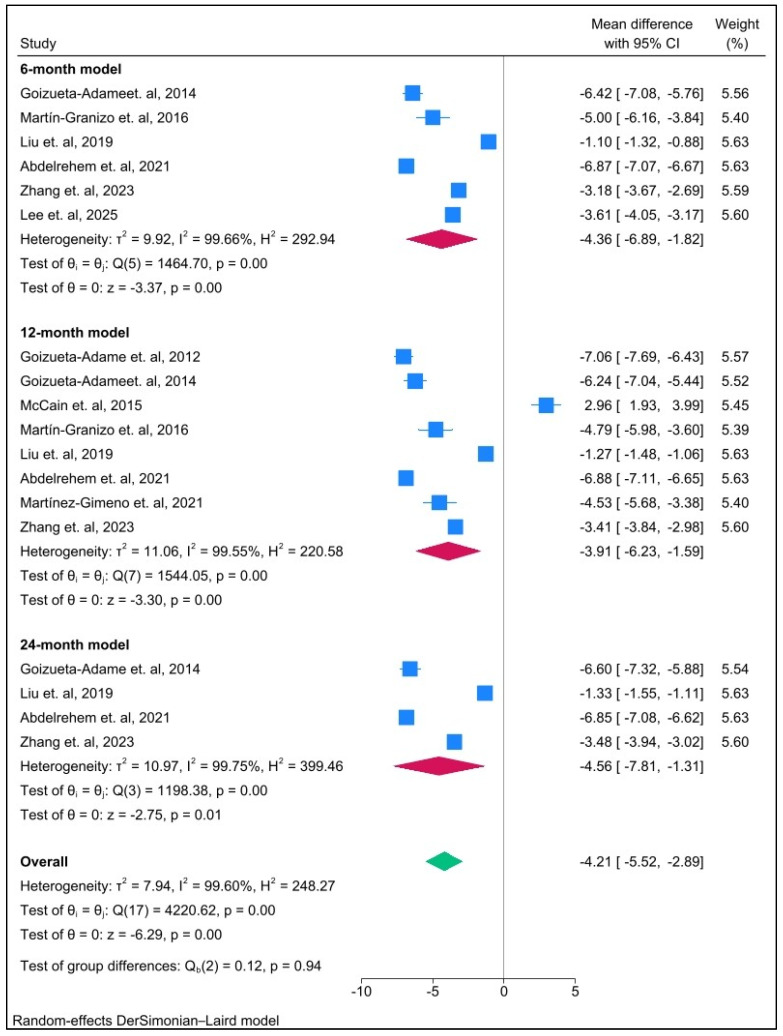
Forest plot illustrating postoperative pain scores (measured by VAS; 0–10 scale) compared with pre-surgery (baseline) across different follow-up time points.

**Figure 5:**
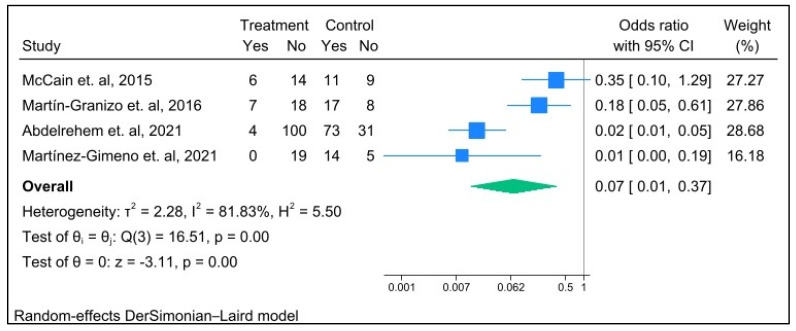
Forest plot illustrating postoperative joint sounds (clicking, popping, or crepitation) compared with pre-surgery (baseline) at the 12-month follow-up.

This technique also presented a highly significant reduction in postoperative pain scores across all follow-up time points. In the short-term (6-month) follow-up period (MD = −4.36; 95% CI: −6.89 to −1.82; *p* ≤ 0.001; I^2^ = 99%), in the intermediate-term (12-month) follow-up period (MD = −3.91; 95% CI: −6.23 to −1.59; *p* ≤ 0.001; I^2^ = 99%) and high significant reduction in the long-term (24-month) follow-up period (MD = −4.56; 95% CI: −7.81 to −1.31; *p* < 0.01; I^2^ = 99%). Moreover, a high level of heterogeneity was observed among the three time points (Figure 4). Although follow-up data on joint sounds were limited in the 12-month follow-up period, this technique also reported a highly significant decrease in the incidence rate of postoperative joint sounds (OR = 0.07; 95% CI: 0.01 to 0.37; *p* < 0.01; I^2^ = 82%) with a high level of heterogeneity (Figure 5).

### 3.5. Assessment of the Quality of Evidence

The quality of evidence for the studies included in the meta-analysis was collectively categorized as low using the GRADE approach (Table 8). This rating was primarily due to heterogeneity across most comparisons and the inclusion of observational studies.

## 4. Discussion

Generally, clinical characteristics of TMD are limited mouth opening, TMJ pain, and joint sounds. Abnormal disc position, especially ADDwoR, has been associated with ID and osteoarthritis. Among stepwise treatment approaches, surgical techniques stand out for functional recovery, pain relief, and long-term joint stability. Nevertheless, open surgery remains controversial due to its invasiveness. Nowadays, there is a growing consensus on the management of ADDwoR. Additionally, most therapeutic approaches have relied on non-surgical treatment, including patient education, physiotherapy, and occlusal splint therapy. More recently, disc repositioning and fixation procedures, especially those performed under arthroscopic guidance, have been increasingly reported as potential treatment options for disc displacement. Therefore, the present systematic review and meta-analysis aimed to evaluate the clinical efficacy of arthroscopically assisted disc repositioning and fixation for the management of anterior disc displacement of the TMJ.

The present systematic review and meta-analysis provide evidence that arthroscopic disc repositioning combined with anchor fixation yields favourable clinical outcomes in patients with TMJ disc displacement. Across short-, intermediate-, and long-term follow-up periods, this surgical approach was consistently associated with significant improvements in MIO, reductions in pain scores, and decreased incidence of joint sounds. These findings support the role of minimally invasive arthroscopic techniques in the management of symptomatic TMJ disc displacement, particularly when non-surgical treatment has failed.

The improvement in MIO was both statistically and clinically significant at all follow-up intervals, with the largest mean difference observed at 24 months. Figure 3 illustrates the postoperative improvement in MIO at 6, 12, and 24 months after surgery, with pooled mean differences exceeding 9 mm across all models. These findings indicate sustained recovery of TMJ function following arthroscopic discopexy [15,16,17,28,29,30,32,33,34].

Figure 4 demonstrates postoperative improvement in pain by VAS at 6, 12, and 24 months after surgery [15,16,17,28,29,30,32,33,34]. A pooled mean difference demonstrated significant decreases with mean reductions of over 3 points in all models. Although a pool mean difference at 12 months after operation illustrated a minimal increase correlated with a slight decrease in MIO, at 24 months, all results show the highest satisfaction across all observation periods. It can be concluded that arthroscopic discopexy techniques improve both function and clinical outcomes in patients with TMJ disc displacement. In contrast, McCain et al. (2015) noted worsened postoperative pain in the successful case patients’ group [16]. This exception highlights that benefits vary across patients and indications warrant careful consideration.

Because joint sound follow-up was limited at 12 months postoperatively, the pooled odds ratio indicated that this operation had a significant impact on the incidence rate of joint sounds (Figure 5) [16,30,33,34]. It can be assumed that the articular disc remains appropriately positioned, and mechanical interference was treated at 12 months. In the future, extended postoperative MRI to assess the position of the TMJ disc would help verify both the clinical effect and the underlying anatomy.

Overall, improvements in MIO, pain, and joint sounds indicate that arthroscopic discopexy can effectively improve TMJ dysfunction. By restoring disc–condyle relationships, these techniques can normalize joint loading and prevent progressive degenerative changes. Nevertheless, due to high heterogeneity following patient selection, surgical expertise, fixation techniques, and postoperative rehabilitation protocols, the interpretation of these results should be performed with caution, as high heterogeneity reduces confidence in the pooled MD estimate. Moreover, the methodological quality was limited in the observational study design, resulting in a low-grade rating. In the future, high-quality randomized trials with standardized protocols should be performed.

Arthroscopic discopexy is a minimally invasive surgical procedure reported to provide early improvement. Although it is classified as Level 3 out of 4 in terms of arthroscopic surgical complexity, the rate of postoperative complications has been reported to be low (ranging from 1.7% to 4.4%) [53,54,55]. The most common complications are otological complications, extravasation of irrigation fluid, and intra-articular bleeding [55,56]. However, these complications can be prevented and managed by having the procedure performed by an experienced surgeon, properly positioning the patient, monitoring irrigation flow and pressure, and avoiding instrument-related iatrogenic injuries [55,56]. Overall, arthroscopic discopexy has benefits over non-surgical treatment by decreasing the long-term recurrence rate and directly treating any pathology, and over open surgery by being a minimally invasive procedure in selected cases [57]. In comparison with the VAS and MIO outcomes reported in the systematic review and meta-analysis of invasive surgical procedures, arthroscopic discopexy demonstrated a more favourable pooled mean difference, despite the limitation of heterogeneous follow-up durations across studies [58]. By limiting disease progression, it can restore normal joint mechanics, reduce abnormal loading, and potentially prevent degeneration. Nevertheless, this review has several limitations. First, the evidence base was limited by the small number of studies, variation in long-term postoperative variables and MRI, and ADD subtype classification [42,59]. Future well-designed RCTs are needed to confirm these findings. Second, designs varied considerably across studies. Third, publication bias is possible, as negative or inconclusive results may not be published.

## 5. Conclusions

Although the level of evidence remains limited, this systematic review and meta-analysis confirm that arthroscopic discopexy was associated with significant improvements in TMJ function, a decrease in joint sounds and chronic discomfort, which compromise quality of life across different follow-up periods. However, due to high heterogeneity and an insufficient evidence base, these results should be interpreted cautiously, and patients should be carefully selected. In the future, larger patient numbers and well-designed RCTs with various observational outcomes are crucial to establish the true efficacy and safety profile of this technique.

## Figures and Tables

**Table 1:** PICOS-based search strategy and terms used across databases (PubMed, Embase, Scopus, and Web of Science.

PICO Question		Search Terms
P	Patients diagnosed with temporomandibular joint disorders, specifically articular disc displacement (with or without reduction, with or without limited mouth opening)	(“Temporomandibular Joint Disorders”[Mesh] OR “Temporomandibular Disorders”[tiab] OR TMD [tiab]) AND (“Temporomandibular Joint Disc Displacement”[Mesh] OR “disc displacement”[tiab] OR “disk displacement”[tiab] OR “anterior disc displacement”[tiab] OR “internal derangement”[tiab])
I	Arthroscopic disc repositioning and anchor fixation techniques (Suturing, pin, or screw)	(“Open Surgical Procedures”[Mesh] OR “open surgery”[tiab] OR “open reduction”[tiab] OR “open joint surgery”[tiab] OR “Arthroscopy”[Mesh] OR arthroscopy[tiab])
C	Pre-surgical baseline values (VAS, MIO, Joint sounds) compared with post-surgical outcomes (before–after comparison)	
O	Pain score by VAS (score 1–10) Maximal interincisal opening (MIO) (mm) Joint sounds (clicking, popping, or crepitation) (Present/Absent)	(“Complications”[Mesh] OR “postoperative complications”[Mesh] OR complication[tiab] OR complications[tiab] OR “adverse event”[tiab] OR “adverse events”[tiab] OR “adverse effects”[tiab] OR “surgical outcome”[tiab] OR outcome[tiab] OR outcomes[tiab] OR sequelae[tiab] OR morbidity[tiab] OR “treatment failure”[tiab])
S	Clinical studies (prospective or retrospective cohort study, case series and randomized controlled trials)	

TMD, Temporomandibular Disorder and VAS, visual analogue scale.

**Table 2:** Inclusion and exclusion criteria.

	Inclusion Criteria	Exclusion Criteria
Language	English	Non-English
Study design	Full-text peer-reviewed clinical studies (RCTs, cohort studies, case series)	Reviews, meta-analyses, abstracts, posters, protocols, books
Population	Patients diagnosed with TMJ disc displacement (with or without reduction)	Animal, cadaveric
Type of operation	Arthroscopic disc repositioning with anchor fixation (suture/pin/screw), or Disc discopexy technique	Other surgical procedures
Surgical site	Temporomandibular joint	Other areas
Outcomes	Reported at least one clinical outcome (Pain score by VAS, MIO, joint sounds)	Studies without relevant clinical outcomes
Timeframe	Studies published up through 1 September 2025	Studies published after the cut-off date

RCTs, Randomized Controlled Trials; TMJ, Temporomandibular joint; VAS, visual analogue scale; MIO, maximal interincisal opening.

**Table 3:** Main characteristics of the included studies.

First Author, Year	Study Design	Patients/ TMJ (n)	Gender (M, F)	Age Range (y), Mean (SD)	Diagnosis	Arthroscopic Discopexy Technique	Resorbable Suture Material	Location or Material of Anchorage	Additional Surgical Procedure and Postoperative Management
Goizueta- Adame et al., 2014 [28]	CS	27/34	M1, F26	17–45, 31.2 ±NM	ADDwR (n = 3) ADDwoR (n = 27) ADDwoR with bone surface change (n = 4)	An arthroscopic disc repositioning using resorbable pin	Not used	Resorbable pin at the lateral surface of the convexity of the condyle (Two pins 33 joints, single pin 1 joints (because small perforation was found)) (SmartNail^®^; PLA)	1. Capsulotomy-myotomy 2. Disc reduction 3. Infiltration with sodium hyaluronate Post-operative management: 1. Medication (lornoxicam, ±muscle relaxant) 2. Functional rehabilitation 3. Soft diet 4. Mouth opening and lateral excursion were not forced for 3 months 5. Occlusal splint
Liu et al., 2019 [29]	RS	532/ 749	M147, F385	13–63, 21.23 ±3.53	Rotational ADD Anterolateral (n = 408) Anteromedial (n = 341)	Modified Yang’s discopexy: double horizontal mattress–like suturing	No (Polyester)	Tragus cartilage (Suture knot)	1. The anterior attachment release 2. Disc reduction Post-operative management: 1. Medication (Antibiotics and NSAID) 2. Soft diet for 3 days 3. Mouth opening exercise start after operation 1 week 4. A functional appliance (in transient postoperative malocclusion)
Abdelrehem et al., 2021 [30]	PS	104/ 130	M22, F82	16–57, 27.47 ±9.65	ADDwR (n = 23) ADDwoR (n = 107)	Yang’s discopexy: double horizontal mattress–like suturing (Yang et al., 2012 [31])	No	The anterior wall of the external auditory canal (Suture knot)	1. The anterior attachment release 2. Disc reduction Post-operative management: NM
Zhang et al., 2023 [17]	RS	24/27	M2, F22	NM, 24.38 ±8.04	Relapsed ADD	The redo arthroscopic disc repositioning and suturing (Single suture discopexy technique at bilaminar zone and posterior band of the disc)	NM	External auditory canal cartilage (Suture knot)	1. The anterior attachment release 2. Disc reduction Post-operative management: 1. Medication (Antibiotics and NSAID for three days) 2. Mouth opening exercise was started after operation 1 week (vertical and lateral step by step for 3–6 months) 3. Stabilizing soft splint for 6 months 4. Orthodontic treatment (if needed)
Lee et al., 2025 [15]	CS	31/31	M2, F29	16–45, 26.35 ±7.26	ADDwoR (Wilkes Classification III-IV)	Posterior double-pass suture technique	No (2/0-Ti-Cron™ polyester)	Subcutaneous tissue (Suture knot)	1. The anterior attachment release 2. Disc reduction Post-operative management: 1. Anterior disc repositioning splint 2. Mouth opening training (for 2–3 months after operation 2 days)
Goizueta- Adame et al., 2012 [32]	CS	16/21	M1, F15	17–49, 32 ±8.00	ADDwR (n = 1) ADDwoR (n = 12) ADDwoR + OA (n = 3)	Posterior double-pass suture technique	Yes (2/0-PDS)	Subcutaneous tissue (Suture knot)	1. Removed adhesion 2. Capsulotomy with anterior myotomy 3. Disc reduction Post-operative management: 1. Medication (Piroxicam, Omeprazole, and Metamizol) 2. Functional rehabilitation 3. Soft diet for at least two months
McCain et al., 2015 [16]	PS	32/42 (s:20 /29)	M4, F28 (s: M2, F18)	Success group: NM, 27.36 ±12.5	Wilkes Classification II-V	Single suture discopexy technique	Yes (0-PDS)	Lateral capsule and Subcutaneous tissue (Suture knot)	1. The anterior attachment release and lavage 2. Complete pterygoid myotomy 3. Retrodiscal contracture or scarification 4. Disc reduction Post-operative management: NM
Martín- Granizo et al., 2016 [33]	CS	26/34	NM	22–65, 41.5 ±NM	ADDwoR (Wilkes Classification III)	An arthroscopic disc repositioning using resorbable pin	Not used	Resorbable pin (SmartNail^®^; PLA) (Two pins 13 joints, single pin 21 joints)	1. The anterior attachment release and lavage 2. Partial pterygoid myotomy 3. Disc reduction Post-operative management: 1. Medication (NSAID) 2. Soft diet for at least one month 3. Active physiotherapy during first month (3 sections per day) 4. Splint therapy
Martínez- Gimeno et al., 2021 [34]	RS	19/21	M1, F18	22–58, 36.84 ±11.16	ADDwR (n = 16) ADDwoR (n = 5)	Single suture discopexy with single-port arthroscope	No (3/0-nylon)	Tragus cartilage (in Antero-posterior direction)	1. Removed intraarticular adhesion (if necessary) 2. No Capsulotomy or anterior myotomy 3. Disc reduction Post-operative management: 1. Medication (Metamizole and Diclofenac) 2. Soft diet and limited mouth opening for one week after operation, then normal diet and mouth opening exercise

NM, not mentioned; s, success group; ADD, Anterior disc displacement; CS, Case series; RS, retrospective cohort, single-arm; PS, Prospective cohort, single-arm; ADDwR, Anterior disc displacement with reduction; ADDwoR, Anterior disc displacement without reduction; OA, Degenerative arthrosis; PDS, Polydioxanone suture; PLA, Polyglycolic polylactic acid; g, gram; NSAID, non-steroidal anti-inflammatory drugs.

**Table 4:** Baseline characteristics and treatment outcome of the included studies.

First Author, Year	MIO (mm) (Mean ± SD)			VAS (0–10) (Mean ± SD)			Joint Sounds (n) Present (P)-Absent (A) Case (%)		
	Pre-surgery	Post-surgery	n	Pre-surgery	Post-surgery	n	Pre-surgery	Post-surgery	n
Follow-up post-surgery 6-month model (Short-term)									
Goizueta-Adame et al., 2014 [28] ^▲▲▲▲^	34.3 ± 5.2 n = 34(NM)	38.4 ± 5.5 n = 29(NM)	-	7.08 ± 1.65 n = 32(NM)	0.66 ± 0.92 n = 29(NM)	-	P: 12 (35.3%) A: 22 (64.7%)	P: NM A: NM	34 (patients)
Martín-Granizo et al., 2016 [33] ***	32.04 ± 7.54	38.14 ± 4.61	26 (patients)	6.58 ± 2.45	1.58 ± 1.75	26 (patients)	NM	NM	NM
Liu et al., 2019 [29]	26.65 ± 7.87	32.68 ± 6.37	532 (patients)	2.06 ± 2.13	0.96 ± 1.56	532 (patients)	NM	NM	NM
Abdelrehem et al., 2021 [30] ^▲▲▲▲^	17.08 ± 2.90	35.61 ± 2.22	104 (patients)	7.05 ± 1.12	0.18 ± 0.38	130 (joints)	P: 73 (70.2%) A: 31 (29.8%)	P: 1 (1.00%) A: 103 (99.00%)	104 (patients)
Zhang et al., 2023 [17] **	25.07 ± 9.73	37.37 ± 6.67	24 (patients)	4.11 ± 0.85	0.93 ± 0.87	24 (patients)	NM	NM	NM
Lee et al., 2025 [15]	23.13 ± 3.54	39.35 ± 2.50	31 (patients)	4.77 ± 0.96	1.16 ± 0.79	31 (patients)	NM	NM	NM
Follow-up post-surgery 12-month model (Mid-term)									
Goizueta-Adame et al., 2012 [32]	31.13 ± 4.63	39.88 ± 2.61	16 (patients)	7.08 ± 1.65 n = 32(NM)	1.00 ± 0.96	16 (patients)	NM	NM	NM
Goizueta-Adame et al., 2014 [28] ^▲▲▲▲^	34.3 ± 5.2 n = 34(NM)	43.2 ± 6.1 n = 29(NM)	-	7.08 ± 1.65 n = 32(NM)	0.84 ± 1.54 n = 29(NM)	-	P: 12 (35.3%) A: 22 (64.7%)	P: NM A: NM	34 (patients)
McCain et al., 2015 [16] *, ^▲▲▲^	30 ± 8	37.9 ± 3.8	20 (patients)	4.19 ± 1.44	7.15 ± 1.87	20 (patients)	P: 11 (61%) A: 9 (49%)	P: 6 (46%) A: 14 (54%)	20 (patients)
Martín-Granizo et al., 2016 [33] ***, ^▲^	32.04 ± 7.54	39.57 ± 4.54	26 (patients)	6.58 ± 2.45	1.79 ± 1.88	26 (patients)	P: 17 (65%) A: 8 (35%)	P: 7 (29%) A: 18 (71%)	25 (patients)
Liu et al.,2019 [29]	26.65 ± 7.87	34.02 ± 6.12	532 (patients)	2.06 ± 2.13	0.79 ± 1.24	532 (patients)	NM	NM	NM
Abdelrehem et al., 2021 [30] ^▲▲▲▲^	17.08 ± 2.90	37.20 ± 1.63	104 (patients)	7.05 ± 1.12	0.17 ± 0.76	130 (joints)	P: 73 (70.2%) A: 31 (29.8%)	P: 4 (3.80%) A: 100 (96.20%)	104 (patients)
Martínez-Gimeno et al., 2021 [34] **, ^▲▲^	36.21 ± 8.09	39.58 ± 4.35	19 (patients)	5.53 ± 1.93	1.00 ± 1.67	19 (patients)	P: 14 (73%) A: 5 (27%)	P: 0 (0%) A: 19 (100%)	19 (patients)
Zhang et al., 2023 [17] **	25.07 ± 9.73	39.43 ± 7.23	24 (patients)	4.11 ± 0.85	0.70 ± 0.67	24 (patients)	NM	NM	NM
Follow-up post-surgery 24-month model (Long-term)									
Goizueta-Adame et al., 2014 [28] ^▲▲▲▲^	34.3 ± 5.2 n = 34(NM)	43.9 ± 4.8 n = 12(NM)	-	7.08 ± 1.65 n = 32(NM)	0.48 ± 0.76 n = 12(NM)	-	P: 12 (35.3%) A: 22 (64.7%)	P: NM A: NM	34 (patients)
Liu et al., 2019 [29]	26.65 ± 7.87	34.73 ± 6.28	532 (patients)	2.06 ± 2.13	0.73 ± 1.43	532 (patients)	NM	NM	NM
Abdelrehem et al., 2021 [30] ^▲▲▲▲^	17.08 ± 2.90	38.18 ± 1.21	104 (patients)	7.05 ± 1.12	0.20 ± 0.78	130 (joints)	P: 73 (70.2%) A: 31 (29.8%)	P: 0 (0%) A: 104 (100%)	104 (patients)
Zhang et al., 2023 [17] **	25.07 ± 9.73	38.44 ± 7.08	24 (patients)	4.11 ± 0.85	0.63 ± 0.79	24 (patients)	NM	NM	NM

n, numbers of participants or TMJs; MIO, Maximal interincisal opening; mm, millimetre; VAS, Visual analogue scale (0–10); P, Present; A, Absent; NM, not mentioned. * Report only successful cases (20 Patients, 29 Joints). ** Unclear unit of n, so researchers assume that unit of n is patients. *** One patient was lost to follow-up and excluded from the study (pre-surgery: n = 26, post-surgery: n = 25). **^▲^** joint sounds (clicking and popping). ^▲▲^ Joint sounds (clicking or joint sounds present). **^▲▲▲^** joint sounds (clicking and crepitation). ^▲▲▲▲^ joint sounds (clicking).

**Table 5:** Summary of arthroscopic disc repositioning and suturing techniques from a systematic review.

First Author, Year	Arthroscopic Discopexy Technique	Anchorage	Included Model or Excluded Result
Israel, H. A., 1989 [35]	Single suture discopexy technique (at posterior lateral corner of the disc-condyle)	Lateral capsule and Subcutaneous tissue (non-resorbable suture). Disc position maintained by retrodiscal scarring after stitch removal (1–2 weeks)	Reported new surgical technique
Tarro et al., 1989 [36]		Lateral capsule and Subcutaneous tissue (Resorbable surgical knot) (holding the disc in a posterior lateral position)	Different follow-up time points (post-operation, 3–12 months)
McCain et al., 1992 [37]			Reported new surgical technique
Zhang et al., 2011 [38]			Different follow-up time points (post-operation $\bar{x}$ = 10.3 months)
McCain et al., 2015 [16]			Included in 12-month model
McNamara et al., 1996 [39]		Cartilaginous part of the external auditory canal (endaural plication) using a non-resorbable suture; the surgical stitch was removed 2 weeks after the operation	Different follow-up time points (post-operation 3 months and 3 years)
Martínez-Gimeno et al., 2021 [34]	Single suture discopexy at the posterolateral disc-condyle corner using a single-port arthroscope (irrigation, visualization, and laser in one working cannula)	Tragal cartilage (anteroposterior direction); non-resorbable surgical knot; stitch removed 2 weeks postoperatively	Included in 12-month model
Rosenbrg and Goss, 2020 [40]	Single suture discopexy technique (at bilaminar zone or retrodiscal zone)	External auditory canal cartilage (non-resorbable suture) (endaural approach); stitch removed 2 weeks postoperatively	Reported new surgical technique
Zhang et al., 2023 [17]	The redo arthroscopic disc repositioning and suturing (Single suture discopexy technique at bilaminar zone and posterior band of the disc)	External auditory canal cartilage (Suture knot)	Included in 6-month, 12-month, and 24-month model
Ward et al., 2025 [18]	Double-suture arthroscopic discopexy (modified from the single-suture discopexy technique)	Posterolateral aspect of the articular disc anchored to the TMJ fibrous capsule, and the posteromedial aspect anchored to the tragal cartilage (resorbable surgical knot)	Different follow-up time points (post-operation $\bar{x}$ = 9 months)
Goizueta-Adame et al., 2012 [32]	Posterior double-pass suture technique, with the needle penetrating the disc from lateral to medial in the posterior zone	Subcutaneous tissue (Resorbable surgical knot)	Included in 12-month model
Lee et al., 2025 [15]		Subcutaneous tissue (non-resorbable surgical knot)	Included in 6-month model
Del Santo et al., 2023 [41]		Tragus cartilage (resorbable surgical knot)	Reported new surgical technique
Zhang et al., 2010 [42]	An arthroscopic double horizontal mattress–like suturing technique performed through 3 punctures (triangulation technique) for pure ADD (Yang’s technique)	The anterior wall of the external auditory canal (non-resorbable surgical knot)	Different follow-up time points (post-operation 1–7 days)
Wang et al., 2011 [43]			Different follow-up time points (post-operation, 49 days)
Yang et al., 2012 [31]			Reported new surgical technique
Abdelrehem et al., 2021 [30]			Included in 6-month, 12-month, and 24-month model
Jerez et al., 2022 [44]	Modified Yang technique (Yang et al., 2012 [31]): five punctures; the suture passes from the posterior border of the bilaminar zone and the posterior band of the disc to the medial retrodiscal tissue in the upper joint space	The anterior wall of the external auditory canal (resorbable or non-resorbable surgical knot)	Reported new surgical technique
de Barros and Ono, 2025 [45]	Modified Yang technique (Yang et al., 2012 [31]): arthroscopic access to both upper and lower compartments	The retrotragal cartilage	Reported new surgical technique
Liu et al., 2019 [29]	Modified Yang’s discopexy: double horizontal mattress–like suturing (6 punctures) for pure ADD/rotational DD; complex technique	Tragus cartilage (Suture knot)	Included in 6-month, 12-month, and 24-month model
Dong et al., 2025 [46]	Modified Yang’s technique: double horizontal mattress–like suturing (5 punctures) for pure ADD modified from Yang et al. (2012) [31] and Liu et al. (2019) [29]	External auditory canal cartilage (non-resorbable surgical knot)	Different follow-up time points (post-operation, 1 and 3 months)
Goizueta-Adame et al., 2014 [28]	An arthroscopic disc repositioning using resorbable pin	Resorbable pin at the lateral surface of the convexity of the condyle (SmartNail^®^; PLA)	Included in 6-month, 12-month, and 24-month model
Martín-Granizo et al., 2016 [33]		Resorbable pin at postero-lateral corner of the disc (SmartNail^®^; PLA)	Included in 6-month and 12-month model
Martín-Granizo et al., 2022 [47]			Reported new surgical technique
Millón-Cruz et al., 2020 [48]			Same subject group with Martín-Granizo et al., 2016 [33]
González-García et al., 2008 [49]	Unclear surgical technique		Unclassified disc discopexy technique
Ângelo et al., 2023 [50]	Unclear surgical technique		Different follow-up time point (post-operation $\bar{x}$ = 523.7 days)
McCain et al., 1992 [37]	-	-	Reports not retrieved
McCain and Menis, 1996 [51]	-	-	Reports not retrieved
McCain and Hossameldin, 2011 [52]	-	-	Reports not retrieved
Tang et al., 2024 [19]	-	-	Systematic review and meta-analysis of Open Discopexy with suture Anchors

TMJ, Temporomandibular joint; ADD, Anterior disc displacement; DD, disc displacement.

**Table 6:** Quality assessment of included study by NHBI Qualifier Score in cohort study design.

First Author, Year	NHLBI Quality Assessment Tool for Before-After (Pre-Post) Study with No Control Group												Quality (Overall Judgement)
	Q1	Q2	Q3	Q4	Q5	Q6	Q7	Q8	Q9	Q10	Q11	Q12	
Liu et al., 2019 [29]	Yes	Yes	Yes	Yes	Yes	Yes	Yes	No	Yes	Yes	Yes	NA	Good (10)
Abdelrehem et al., 2021 [30]	Yes	Yes	Yes	Yes	Yes	Yes	Yes	No	Yes	Yes	Yes	NA	Good (10)
Zhang et al., 2023 [17]	Yes	Yes	Yes	Yes	NR	Yes	Yes	No	Yes	Yes	Yes	NA	Good (9)
McCain et al., 2015 [16]	Yes	Yes	No	No	NR	Yes	Yes	No	NR	No	No	NA	Poor (4)
Martínez-Gimeno et al., 2021 [34]	Yes	Yes	Yes	Yes	NR	Yes	Yes	No	Yes	Yes	Yes	NA	Good (9)

Yes, the study meets the criterion; No, the study does not meet the criterion; NA (Not Applicable), The item is not relevant to the particular study design; NR, not reported. Q1: Was the study question or objective clearly stated? Q2: Were eligibility/selection criteria for the study population prespecified and clearly described? Q3: Were the participants in the study representative of those who would be eligible for the test/service/intervention in the general or clinical population of interest? Q4: Were all eligible participants who met the prespecified entry criteria enrolled? Q5: Was the sample size sufficiently large to provide confidence in the findings? Q6: Was the test/service/intervention clearly described and delivered consistently across the study population? Q7: Were the outcome measures prespecified, clearly defined, valid, reliable, and assessed consistently across all study participants? Q8: Were the people assessing the outcomes blinded to the participants’ exposures/interventions? Q9: Was the loss to follow-up after baseline 20% or less? Were those lost to follow-up accounted for in the analysis? Q10: Did the statistical methods examine changes in outcome measures from before to after the intervention? Were statistical tests performed that provided *p*-values for the pre-to-post changes? Q11: Were outcome measures of interest taken multiple times before the intervention and multiple times after the intervention (i.e., did they use an interrupted time-series design)? Q12: If the intervention was conducted at a group level (e.g., a whole hospital, a community, etc.) did the statistical analysis take into account the use of individual-level data to determine effects at the group level?

**Table 7:** Quality assessment of the included study by the NHBI Qualifier Score in the case series study design.

First Author, Year	NHLBI Quality Assessment Tool for Case Series Studies									Quality (Overall Judgement)
	Q1	Q2	Q3	Q4	Q5	Q6	Q7	Q8	Q9	
Goizueta-Adame et al., 2012 [32]	Yes	Yes	NR	Yes	Yes	Yes	Yes	Yes	Yes	Good (8)
Goizueta-Adame et al., 2014 [28]	Yes	Yes	Yes	Yes	Yes	Yes	Yes	Yes	No	Good (8)
Lee et al., 2025 [15]	Yes	Yes	NR	Yes	Yes	Yes	Yes	Yes	Yes	Good (8)
Martín-Granizo et al., 2016 [33]	Yes	Yes	NR	Yes	Yes	Yes	Yes	Yes	Yes	Good (8)

Yes, the study meets the criterion; No, the study does not meet the criterion; NA (Not Applicable), The item is not relevant to the particular study design; NR, not reported. Assessment: Good: Met 7–9 criteria, Fair: Met 4–6 criteria, Poor: Met 0–3. Q1: Was the study question or objective clearly stated? Q2: Was the study population clearly and fully described, including a case definition? Q3: Were the cases consecutive? Q4: Were the subjects comparable? Q5: Was the intervention clearly described? Q6: Were the outcome measures clearly defined, valid, reliable, and implemented consistently across all study participants? Q7: Was the length of follow-up adequate? Q8: Were the statistical methods well-described? Q9: Were the results well-described?

**Table 8:** Grade assessment result.

Certainty Domain	Justification	Downgrade/Upgrade	Estimation
Start	Observational studies (cohort and case series)	-	Low (⊕⊕◯◯)
Downgrade estimation			
Risk of Bias	Uncontrolled confounding, lack of blinding	Downgraded 1 level	very low (⊕◯◯◯)
Inconsistency	Although heterogeneity was high, the direction of effect was consistent across studies	No downgrade	very low (⊕◯◯◯)
Indirectness	Population, intervention, comparator, and outcomes directly addressed the review question	No downgrade	very low (⊕◯◯◯)
Imprecision	95% CI do not cross the line of no effect; however, intervals were wide in several analyses	No downgrade	very low (⊕◯◯◯)
Publication bias	Not assessed	No downgrade	very low (⊕◯◯◯)
Overall (after downgrades): Very low (⊕◯◯◯)			
Upgrade estimation			
Large Effect	Large effect size	Upgraded 1 level	low (⊕⊕◯◯)
Dose–response	Not observed	No upgrade	low (⊕⊕◯◯)
Residual confounding toward the null	Not observed	No upgrade	low (⊕⊕◯◯)
Final certainty rating: Low (⊕⊕◯◯)			

## Data Availability

No new data were created or analyzed in this study.

## Supplementary Materials

The following supporting information can be downloaded at https://www.mdpi.com/article/10.3390/jcm14228046/s1. Table S1. Full search strategies used in the systematic review and meta-analysis. Supplementary File S1: Preferred Reporting Items for Systematic reviews and Meta-Analyses extension for Scoping Reviews (PRISMA-ScR) Checklist [60].
