# Supplementary material for: Arthroscopic Discopexy Techniques for Articular Disc Displacement: A Systematic Review and Meta-Analysis
Source: J Clin Med. 2025 Nov 13;14(22):8046. doi: 10.3390/jcm14228046 (PMC12653788; doi:10.3390/jcm14228046)
Supplement: Supplementary file 1 [file jcm-14-08046-s001.zip › jcm-3940908-sm.pdf]

Datbase	Search date	Search strategy
1. PubMed<br>(n=101)	September 1st, 2025	(("Temporomandibular Joint Disorders"[Mesh] OR "Temporomandibular Disorders"[tiab] OR TMD[tiab])) AND (("Temporomandibular Joint Disc Displacement"[Mesh] OR "disc displacement"[tiab] OR "disk displacement"[tiab] OR "anterior disc displacement"[tiab] OR "internal derangement"[tiab]))<br><br>AND (("Open Surgical Procedures"[Mesh] OR "open surgery"[tiab] OR "open reduction"[tiab] OR "open joint surgery"[tiab] OR "Arthroscopy"[Mesh] OR arthroscopy[tiab]))<br><br>AND (("Complications"[Mesh] OR "postoperative complications"[Mesh] OR complication[tiab] OR complications[tiab] OR "adverse event"[tiab] OR "adverse events"[tiab] OR "adverse effects"[tiab] OR "surgical outcome"[tiab] OR outcome[tiab] OR outcomes[tiab] OR sequelae[tiab] OR morbidity[tiab] OR "treatment failure"[tiab]))
2. Embase<br>(n=123)	September 1st, 2025	('temporomandibular joint disorder'/exp OR 'temporomandibular disorder':ti,ab OR TMD:ti,ab)<br><br>AND ('temporomandibular joint disk displacement'/exp OR 'disc displacement':ti,ab OR 'disk displacement':ti,ab OR 'anterior disc displacement':ti,ab OR 'internal derangement':ti,ab)<br><br>AND ('open surgery':ti,ab OR 'open reduction':ti,ab OR 'open joint surgery':ti,ab OR arthroscopy:ti,ab OR 'arthroscopy'/exp)<br><br>AND ('complication'/exp OR complication:ti,ab OR complications:ti,ab OR 'adverse event':ti,ab OR 'adverse events':ti,ab OR 'adverse effect':ti,ab OR 'adverse effects':ti,ab OR 'surgical outcome':ti,ab OR outcome:ti,ab OR outcomes:ti,ab OR sequelae:ti,ab OR morbidity:ti,ab OR 'treatment failure':ti,ab)
3.Scopus<br>(n=156)	September 1st, 2025	TITLE-ABS-KEY ("temporomandibular joint disorders" OR "temporomandibular disorders" OR TMD)

		AND TITLE-ABS-KEY ("temporomandibular joint disc displacement" OR "disc displacement" OR "disk displacement" OR "anterior disc displacement" OR "internal derangement")<br><br>AND TITLE-ABS-KEY ("open surgery" OR "open reduction" OR "open joint surgery" OR arthroscopy)<br><br>AND TITLE-ABS-KEY (complication OR complications OR "adverse event" OR "adverse events" OR "adverse effect" OR "adverse effects" OR "surgical outcome" OR outcome OR outcomes OR sequelae OR morbidity OR "treatment failure")
4.Web of Science (n=53)	September 1st, 2025	TS=("Temporomandibular Joint Disorders" OR "Temporomandibular Disorders" OR TMD)<br><br>AND TS=("Temporomandibular Joint Disc Displacement" OR "disc displacement" OR "disk displacement" OR "anterior disc displacement" OR "internal derangement")<br><br>AND TS=("Open Surgical Procedures" OR "open surgery" OR "open reduction" OR "open joint surgery" OR "Arthroscopy" OR arthroscopy)<br><br>AND TS=("Complications" OR "postoperative complications" OR complication OR complications OR "adverse event" OR "adverse events" OR "adverse effects" OR "surgical outcome" OR outcome OR outcomes OR sequelae OR morbidity OR "treatment failure")

Supplemental Table S1. Full search strategies used in the systematic review and meta-analysis.
